# A Stumbling Block

**Published:** 1879-09

**Authors:** 


### Editorial.

### A STUMBLING BLOCK.

Faulty, bungling, and insufficient operations upon the teeth, by men claiming a high order of ability, and who have had opportunity for making good attainments, constitute one of the most serious embarrassments that stand in the way of the best interests of the dental profession. A case or two in illustration, may be given. A few days ago a patient came for treatment of the gums, filling teeth, etc. Attention was first directed to the first superior molar of the right side, and the adjoining bicuspid, about these the gum was swollen almost in a fungus state, and very sensitive, bleeding at the slightest touch. Quite a large cavity on the anterior proximate surface of the molar, had been filled with gold about two years before. The filling now, instead of being in position in the cavity, was a comparatively soft—or rather uncondensed mass of gold packed between the nicks of the teeth; irritating the tissue by pressure, and by retaining foreign matter there also. The gum wasso sensitive that the teeth had not been used for mastication. Since the *stuffiing* had been done, the adjoining teeth had a considerable amount of decaying, and very offensive matter upon them that was injuring the teeth, and the gums about them. With this state of things the patient was greatly annoyed and disgusted, and in addition, suffering all the while. She was rather inclined to regard all dentists as inefficient, and possibly something worse. She had supposed herself to, be in the hands of one of the most skillful. She said "he ought to be, for he has been in practice over twenty-five years."

The treatment in this case was; the removal of the offensive gold, a thorough cleansing of the teeth, depleting the gums, and rubbing them three or four times daily with the finger, and using twice daily an astringent and disinfecting sh. Within a day or two the molar and bicuspid were filled, and from that time they were used in mastication without the slightest discomfort, and the gums are now in a healthy condition.

The patient came with the full belief that the pulp of the tooth was exposed, and just upon the point of aching. There was no exposure, and not even sensitiveness of the dentine. The whole trouble was due to the pressure of the mass of gold upon the tissues between the teeth; which was a result of the criminal ignorance and incompetency, or carelessness of the operator.

Another case, from the same hands, but another patient, who presented an inferior second molar, right side. About three years previous, a large cavity in the buccal surface of this tooth had been filled with gold. The gum along the buccal surface of this, and the adjoining teeth, was much enlarged, sensitive, spongy and bleeding at a touch; the gum covered a large part of the buccal surface of the tooth, and the upper edge of the gold filling was just exposed to view. There was no salivary calculus on these teeth, but some foreign matter, such as would lodge upon teeth that were not used, and the gums so sensitive, that the brush could not be applied. The teeth had not been used in mastication since shortly after the filling had been made. The case seemed to be an obscure one; upon pressing down the margin of the gum, the part of the filling exposed to view, made rather a good showing, though evidently had not been very thoroughly condensed; but upon passing an instrument to the lower border of the filling, it was found protruding fully two-thirds of a line, presenting a rough, ragged edge to the gum that was in contact with it, thus causing irritation, and this was no doubt increased by the accumulation of vitiated matter held under this projection. This was shown to be the cause of the disease of the gum, from the fact that when the filling was dressed and finished, as it should have been in the first place, the affected part was rapidly restored to health. In this case, the presumption is that the difficulty arose from culpable carelessness. Which is the worse, incompetency, or habitual carelessness? Who suffers by such practice, as these cases present?

First, and especially the patient, and in two ways, first, by the disease and pain inflicted, and second, by destroying faith and confidence in the ability of dentists to prevent, mitigate, or lighten disease and suffering.

Second. The whole profession suffers degradation, in the estimation of the public.

Third. The inefficient practitioner always suffers, and in many ways, he usually pays a penalty fully equal to all he gains by his faulty practice.,

Oh! that every dentist in the country, could have a full and just appreciation of the responsibility that rests upon him.

